# An artificial intelligence-enabled ECG algorithm for identifying ventricular premature contraction during sinus rhythm

**DOI:** 10.1186/s40001-022-00929-z

**Published:** 2022-12-14

**Authors:** Sheng-Nan Chang, Yu-Heng Tseng, Jien-Jiun Chen, Fu-Chun Chiu, Chin-Feng Tsai, Juey-Jen Hwang, Yi-Chih Wang, Chia-Ti Tsai

**Affiliations:** 1 Division of Cardiology, Department of Internal Medicine, National Taiwan University College of Medicine and Hospital Yun-Lin Branch, Dou-Liu City, Taiwan; 2 Graduate Institute of Biomedical Electronics and Bioinformatics, National Taiwan University, Taipei, Taiwan; 3 Division of Cardiology, Department of Internal Medicine, Chung Shan Medical University Hospital, Taichung, Taiwan; 4 Division of Cardiology, Department of Internal Medicine, National Taiwan University College of Medicine and Hospital, Taipei, Taiwan; 5 Bioinformatics and Biostatistics Core, Center of Genomic and Precision Medicine, National Taiwan University, Taipei, Taiwan

**Keywords:** Artificial intelligence, Convolutional neural network, 12-Lead electrocardiogram, Ventricular premature complex

## Abstract

**Background:**

Ventricular premature complex (VPC) is a common arrhythmia in clinical practice. VPC could trigger ventricular tachycardia/fibrillation or VPC-induced cardiomyopathy in susceptible patients. Existing screening methods require prolonged monitoring and are limited by cost and low yield when the frequency of VPC is low. Twelve-lead electrocardiogram (ECG) is low cost and widely used. We aimed to identify patients with VPC during normal sinus rhythm (NSR) using artificial intelligence (AI) and machine learning-based ECG reading.

**Methods:**

We developed AI-enabled ECG algorithm using a convolutional neural network (CNN) to detect the ECG signature of VPC presented during NSR using standard 12-lead ECGs. A total of 2515 ECG records from 398 patients with VPC were collected. Among them, only ECG records of NSR without VPC (1617 ECG records) were parsed.

**Results:**

A total of 753 normal ECG records from 387 patients under NSR were used for comparison. Both image and time-series datasets were parsed for the training process by the CNN models. The computer architectures were optimized to select the best model for the training process. Both the single-input image model (InceptionV3, accuracy: 0.895, 95% confidence interval [CI] 0.683–0.937) and multi-input time-series model (ResNet50V2, accuracy: 0.880, 95% CI 0.646–0.943) yielded satisfactory results for VPC prediction, both of which were better than the single-input time-series model (ResNet50V2, accuracy: 0.840, 95% CI 0.629–0.952).

**Conclusions:**

AI-enabled ECG acquired during NSR permits rapid identification at point of care of individuals with VPC and has the potential to predict VPC episodes automatically rather than traditional long-time monitoring.

## Introduction

Ventricular premature complex (VPC), also known as ventricular extrasystole, is a commonly encountered arrhythmia worldwide [1]. According to the previous studies, the prevalence of VPC is around 1–4% in the general populations on standard 12-lead electrocardiography (ECG) [2]. Additionally, increasing age, male gender, atherosclerosis, hypertension, and cardiomyopathy are related to higher occurrence of VPC [1]. Clinically, VPC without any symptoms have been seemed to be benign. However, frequent VPC attacks are associated with cardiomyopathy and irreversible pathogenesis [3]. Especially for those with structurally heart diseases, the incidence and complexity of VPC also increase, up to 90% in ischemic cardiomyopathy [2]. Thence, VPC seems to be the signals for increasing risk of sudden death or the clues for underlying cardiomyopathy. Consequently, timely prediction and intervention of VPC attack might eliminate its arrhythmogenic source and reverse progressive cardiomyopathy.

Clinically, the conventional 12-lead electrocardiogram (ECG) has been used to monitor cardiac structure and physiological condition for decades. ECG is non-invasive, easy to use, rapid, low cost in the resource setting, and simple for interpretations [4]. Due to these characteristics, several ECG monitoring systems are exploited to analyze the signals of ECG [4]. In order to interpret these enormous amount data immediately, deep learning has been widely used to read ECG signals and artificial intelligence (AI) technique is suitable to process countless ECG signals without human intervention and offer accurate diagnoses automatically [4].

However, most of the patients present with intermittent VPC and occasionally all the ECG-related examinations or monitoring are negative for the definite diagnosis of VPC. We need a tool to identify patients with VPC using ECG during sinus rhythm. It has been shown that AI-enabled ECG algorithm can identify patients with paroxysmal atrial fibrillation using ECG during sinus rhythm. In this study, we used the automatic deep-learning neural network to identify the high-risk VPC populations using their ECGs during sinus rhythm for VPC attack to facilitate point of care and hope to prevent severe cardiovascular events in advance.

## Methods

### Data collection and parsing

The data were collected from patients with the diagnosis of VPC at the National Taiwan University Hospital, Taipei, Taiwan from Jan/2021 to Oct/2021. Initially, 398 patients were enrolled and 2515 ECG records were checked. Only ECG during sinus rhythm without the diagnoses of VPC was parsed and finally 1617 ECG records were double-checked by two cardiologists and labeled as sinus rhythm from patients with VPC. For the control group, 1053 patients with 2090 ECG records were collected and screened. Finally, 753 normal ECG records from 387 patients were picked up and marked as normal sinus rhythm (NSR). This study was approved by the ethics committee and institutional review board (IRB) on human research of the Medical Research Department of National Taiwan University Hospital, Taipei, Taiwan (IRB NO: 201705122RINC) and informed consent was waived because identification data on ECGs were removed before they were sent for analyses.

### Dataset preparation

The datasets were divided into the training set, validation set, and test set. First, 50 ECG records were chosen randomly for the validation set and another 100 ECG records were selected for the test set. The rest of the data were assigned to the training set. Importantly, the data of the same patient could not belong to more than one dataset, otherwise, it would affect the credibility of the final results.

### Data type and pre-process

**Fig. 1:**
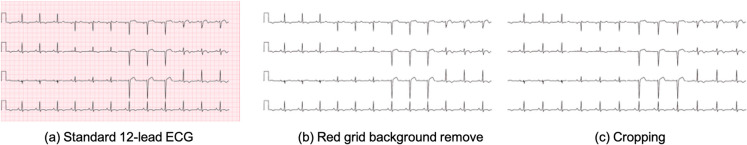
The ECG image processing process before input. **a** The standard 12-lead ECG image. **b** The red-rid background of the 12-lead ECG images was removed. **c** The image was cropped to be focused on the ECG signals

The ECG records collected were in the format of standard 12-lead ECG images, including lead I, II, III, V1 ~ 6, aVR, aVL, aVF, and long lead II (MAC2000 resting ECG System, GE Healthcare). All the records were measured at the frequency of 500 Hz and duration was 2.5 s. Before data analysis, the red-grid backgrounds of the ECG images were removed and coped to make the whole images to be precisely focused on the ECG signals (Fig. 1).

**Fig. 2:**
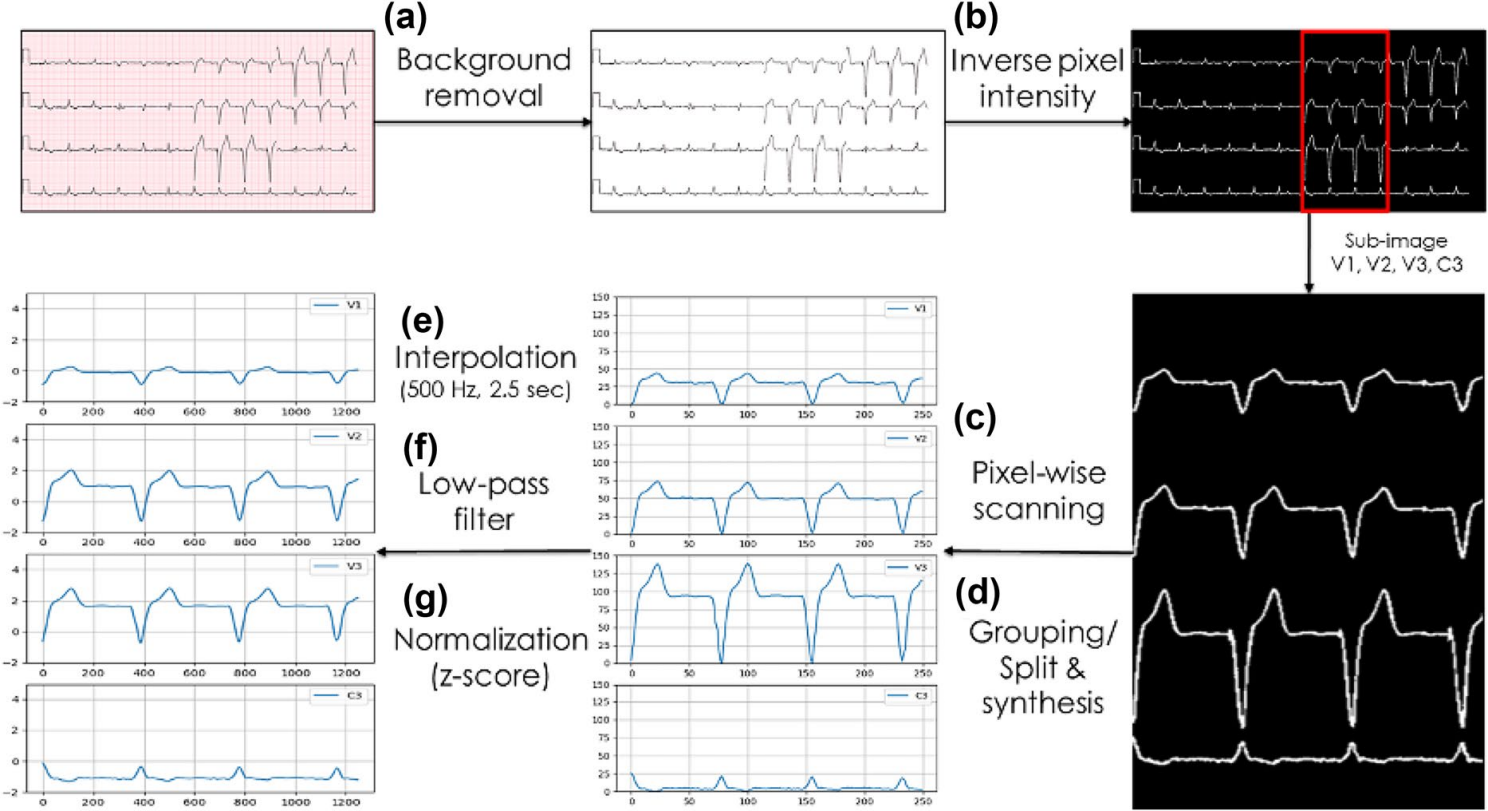
The ECG data input format. **a** The red-grid background of the 12-lead ECG image was removed and ECG was converted to a gray-scale image. **b** The pixel intensity was inversed and the pixel intensity was made to 255 pixels. The image was cut vertically into four sub-images according to the “start” and “end” position of each lead. **c** Pixel-wise scanning sub-images and recording the position where the pixel intensity was equal to 255 pixels. **d** The closest position of the signal was grouped. Each column was split into four values and all values of the columns were synthesized into four lists in each lead. The signals were transferred to be the time-series formats. **e** The column of each sub-image consisted of 250 pixels. After pixel-wise scanning, one lead with 250 time-series data was formatted. The interpolation operations were used to perform up-sampling for the time-series data (500 Hz, 2.5 s). **f** The IIR low-pass filter was used to filtered the noise (cut-off frequency = 15 Hz, order = 3). **g** The magnitude of each lead was normalized into a unified scale

After that, the ECG images were adjusted to be 512 × 256 × 3 pixels. The two-dimensional ECG images were converted into the one-dimensional and time-series data. The input data size was 1250 × 12 pixels for convolutional neural network (CNN) to perform the image recognition (Fig. 2).

### Models process

**Fig. 3:**
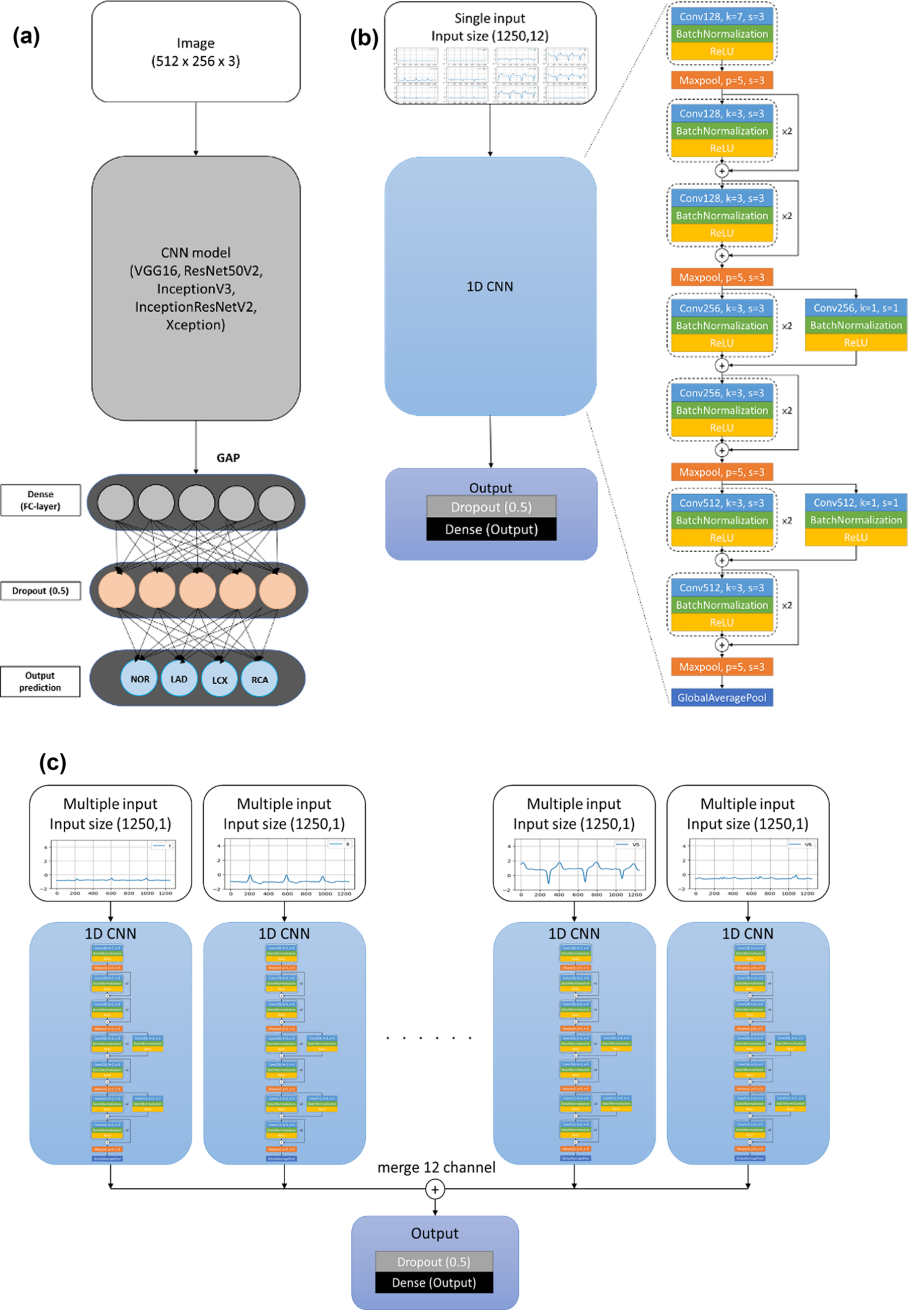
The architecture of the convolutional neural network (CNN). **a** The 2-dimensional image data were processed by five network computer architectures, which included VGG16, ResNet50V2, InceptionV3, InceptionResNetV2, and Xception to get the best image recognition at the Image Net part of CNN and then flattened into Global Average Pooling (GAP). **b** The dense layer of the single input from the 2-dimensional image data was connected. The dropout was added to avoid overfitting (drop rate = 0.5) and another dense layer with size of two was added to get an output layer. *VPC* ventricular premature complex, *NOR* normal rhythm. **c** The signals of time-series data were extracted through the CNN layers and flattened by GAP. The output features of the single -input model was directly connected to dropout (dropout rate = 0.5) and the multiple-input model from the twelve channels’ features were merged (to get the output result (dense size = 2). *GAP* Global Average Pooling

We set up CNN models according to the dimensional characteristics of the data formats. For the 2-dimensional image data, we used five network computer architectures, including VGG16 [5], ResNet50V2 [6], InceptionV3 [7], InceptionResNetV2 [8], and Xception [9] to get the best image recognition with the Image Net part of CNN (Fig. 3a). After the features of the image data were extracted by CNN, the signals were flattened by Global Average Pooling (GAP) [10] and another dense layer was connected. Dropout was added to avoid overfitting later on (drop rate = 0.5) (Fig. 3b) [11]. Finally, another dense layer with a size of two was added, which represented two-type results as output layer (VPC and NSR) (Fig. 3b). For the time-series data, we used single-input and multiple-input computer architectures for the models processing. Initially, we changed the convolutional kernel into a one-dimensional kernel and different kernel sizes were tried by the CNN. The stride was set to three and the moving window of the convolutional kernel spans three grids at once. Each convolutional block was composed of one-dimensional CNN activation by BatchNormalization [12] and ReLU [13]. The setting of Maxpooling [14] was pooling size equal to 5 and stride equal to 3. After the signals of features were extracted through the CNN layers, they were flattened by GAP. The output features of the single-input model were directly connected to dropout (dropout rate = 0.5) to avoid overfitting (Fig. 3c). On the other hand, the multiple-input model merged twelve channels’ features together and connected to one dense layer (dense size = 2) to get the output result (Fig. 3c).

### Training process

We used Google Colaboratory (Colab) [15] with high-Random Access Memory Graphics Processing Unit environment as the training platform. This Colab was supported by the Python 3.8 and Tensorflow package [16] for CNN training process. We also used the keras Application Programming Interface (API) (one deep-learning API written in Python) to build CNN models and ImageNet competition for transferring and learning. The settings of the APIs and the training parameters are shown in Table 1.

**Table 1:** Application programming interfaces and parameter setting

API or Parameters	Name	Setting
Callback	EarlyStopping	Patience = 250
Optimizer	Adam	Learning rate = 0.0011
Metrics	Accuracy	–
Losses	Categorical Crossentropy	–
Epochs	–	400
Batch size	–	32

*API* Application Programming Interface

### Statistical analysis

Optimal cut-points and measurements of diagnostic performance included accuracy, sensitivity, specificity, positive predictive value, negative predictive value, and area under the curve (AUC) of the receiver operating characteristic curve (ROC). All were reported with 2-sided 95% confidence interval. The data were analyzed by IBM SPSS (Version 25 for Windows, Armonk, New York) for statistical analysis.

## Results

### Performance of the image-input model

**Fig. 4:**
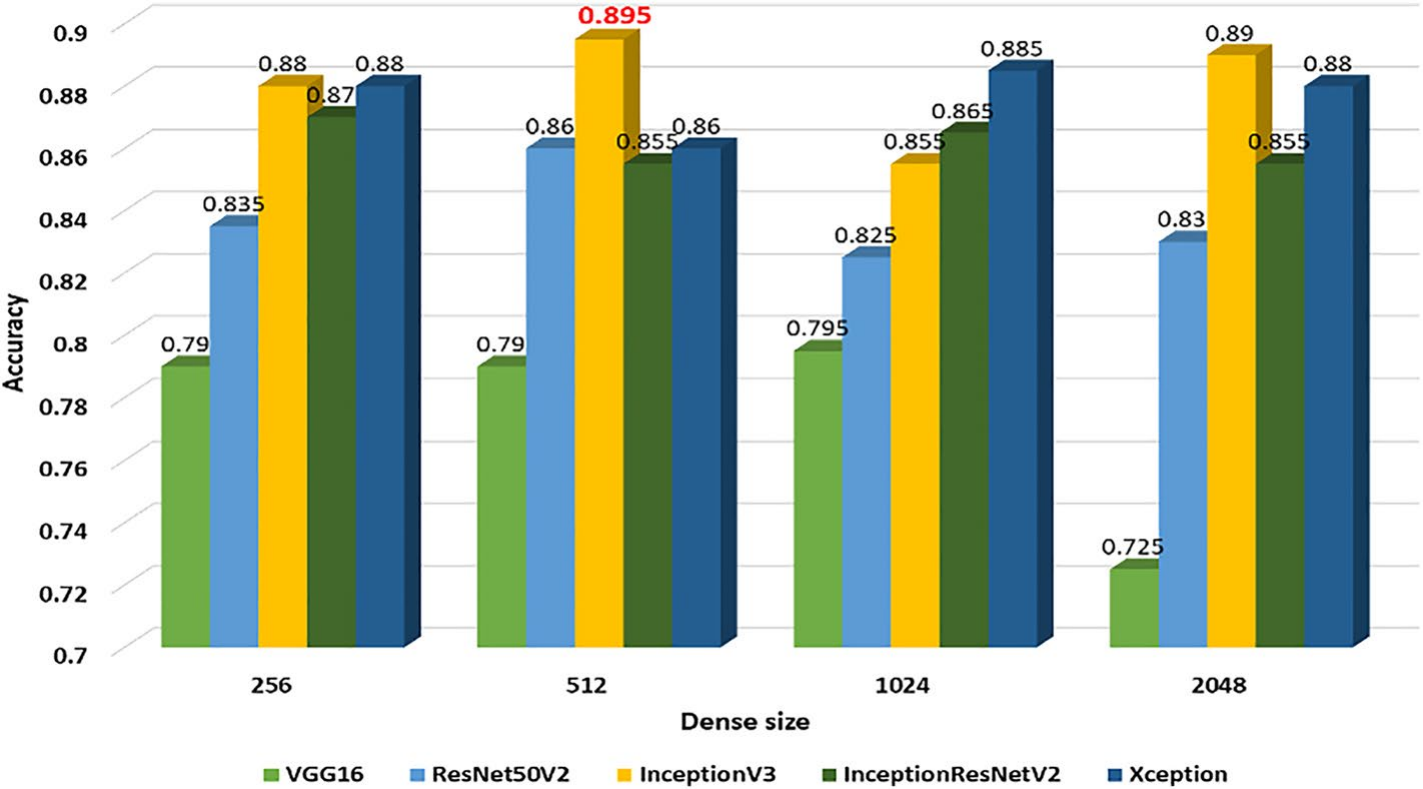
The accuracy of different image-input CNN models with various sizes of connected layers. *CNN* convolutional neural network

**Fig. 5:**
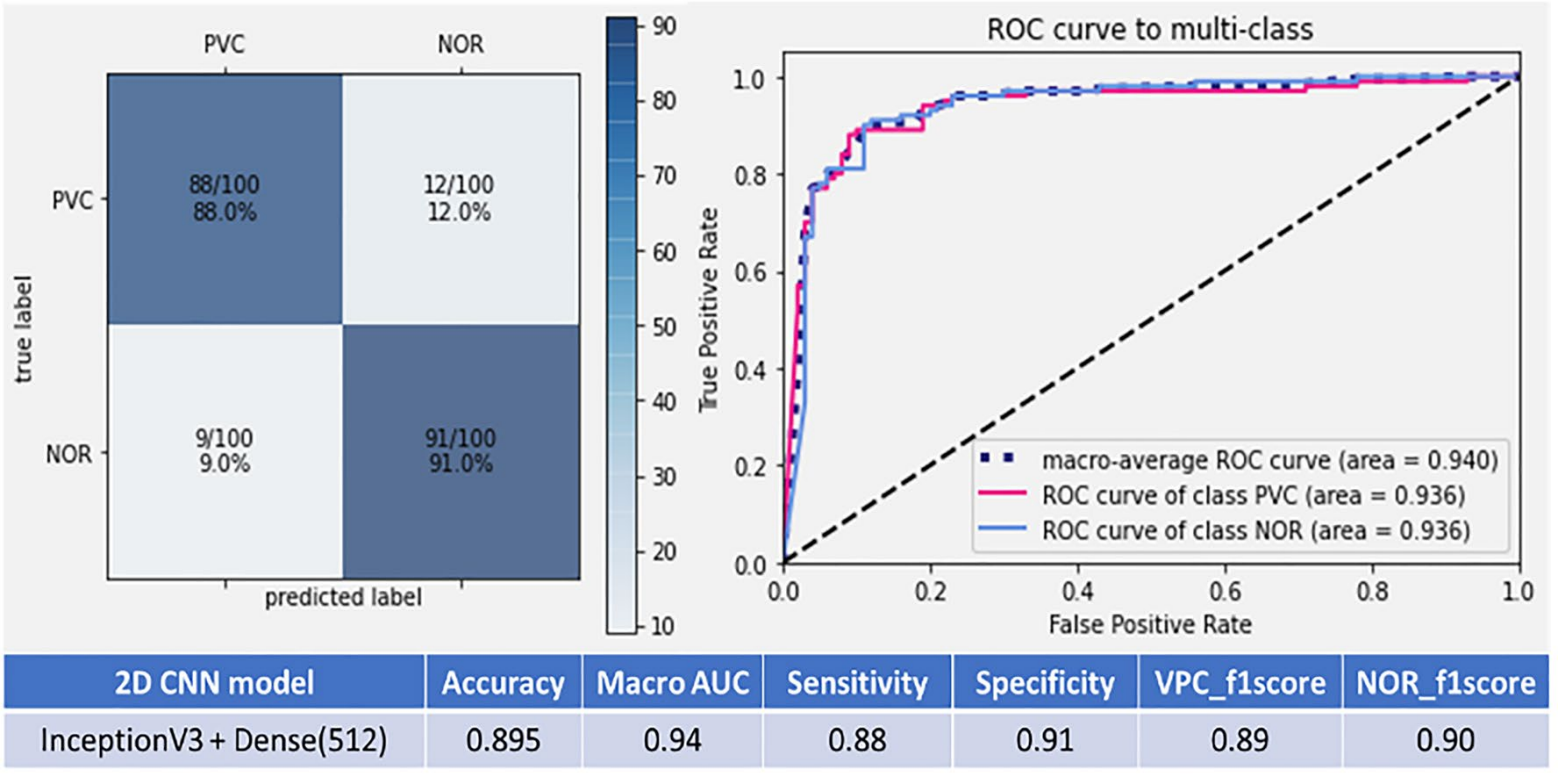
The AUC of the ROC for the combination of the InceptionV3 CNN model and the dense layer (size = 512). *CNN* convolutional neural network, *AUC* area under the receiver operating characteristic curve, *ROC* receiver operating characteristic curve

Among all included patients, the mean age was 62.4 years (standard deviation 14.3) on the date of the first ECG, and 750 (52%) patients were men. In this study, we used different test sets to evaluate the different pre-trained CNN models with various sizes of the dense and the fully connected layers. The five network computer architectures including VGG16 [5], ResNet50V2 [6], InceptionV3 [7], InceptionResNetV2 [8], and Xception [9] were used to choose the best model with the highest accuracy for the following training process. Eventually, the InceptionV3 [7] of the CNN model connected with the dense layer (size = 512) was chosen as the core CNN model for the image format datasets. The accuracy was the highest in comparison with the other combinations (accuracy = 0.895, sensitivity = 0.907, and specificity = 0.883, 95% CI) (Fig. 4). The AUC of the ROC for this model architecture was 0.941 (Fig. 5).

### Performance of the time-series-input model

For the time-series data, we evaluated different sizes of convolution kernels to find the best combination. The best kernel size was 7 to perform the single-input model and 11 for the multi-input model (Table 2). In the multi-input model, the CNN channel needed to analyze the signals of all the twelve leads at the same time. The complexity was relatively higher than that of the single-input model which just needed to analyze one-lead signal. Additionally, the multi-input model used parallelization of analysis. Therefore, the accuracy of the multi-input model was 4% higher than the single-input model (single-input model: 0.840 and multi-input model: 0.880, 95% CI) (Table 3). The accuracy of the multi-input time-series model was still lower than but very close to that of the image-input model (0.880 vs. 0.895).

**Table 2:** Time-series data for the single and multi-input models

Kernel size	Accuracy	ROC AUC	Sensitivity	Specificity
Single-input (1250 × 12)				
3	0.795	0.866	0.798	0.792
5	0.815	0.886	0.889	0.765
7	0.840	0.889	0.886	0.804
9	0.835	0.893	0.885	0.796
11	0.835	0.895	0.868	0.807
Multi-input (1250 × 1) × 12				
3	0.865	0.920	0.884	0.848
5	0.875	0.928	0.895	0.857
7	0.860	0.929	0.875	0.846
9	0.850	0.920	0.917	0.802
11	0.880	0.929	0.896	0.865

*ROC AUC* area under the receiver operating characteristic curve, *VPC* ventricular premature complex

**Table 3:** Comparison of both single-input and multi-input models

Data type	Model	Acc	ROC AUC	Sensitivity	Specificity
Time series	*ResNet50V2 *(Single-input)	0.840	0.889	0.886	0.804
Time series	*ResNet50V2 *(Multi-input)	0.880	0.929	0.896	0.865

*Acc* accuracy, *ROC AUC* area under the receiver operating characteristic curve, *VPC* ventricular premature complex

## Discussion

### Highlights

In this study, our AI model enabled to record ECG signals and detect the presence of VPC during normal sinus rhythm (AUC: 0.941). The accuracy was comparable with a previous study using AI-enable ECG to identify AF during normal sinus rhythm (AUC: 0.87; 95% CI: 0.86–0.88) [17] and were better with other medical screening tests such as CHADS2 score (AUC: 0.64; 95% CI: 0.56–0.72 and CHA2DS2-VASc score (AUC: 0.67; 95% CI, 0.60–0.74) for prediction of ischemic strokes [18].

### The importance for VPC detection during sinus rhythm

Although VPC seems to be benign, it is associated with increasing cardiovascular events. From the Framingham Heart study [19], the Multiple Risk Factor Intervention Trial (MRFIT) [20], and the Atherosclerosis Risk in Communities Study (ARIC) studies [21], VPC has been demonstrated as an independent risk factor for mortalities of the patients without structural heart diseases [1]. VPC is also recognized to trigger ventricular tachycardia/fibrillation and cause sudden cardiac death (SCD) or unexplained syncope in patients without ischemic cardiomyopathy [1]. Additionally, patients with frequent VPCs (defined as > 1 VPC on a 10-s ECG or > 30 VPCs in an hour) are associated with incent heart failure and sudden cardiac death [1]. Besides, patients with frequent VPCs are risky to suffer from VPC-induced cardiomyopathy even though they are asymptomatic [1].

The ability to identify undetected VPC with an inexpensive, widely available, point-of-care test—an ECG recorded during normal sinus rhythm—has important practical implications, particularly for VPC screening efforts or for the management of patients with unexplained syncope or chest discomfort, especially for those with a familial history of SCD. This study shows the power of leveraging modern computing technology, large datasets, non-linear models, and automated features extraction using convolution layers to potentially improve diagnosis and treatment of a disease with a life-threatening state. When VPC is found, treatment could be initiated early. Catheter ablation significantly improves the outcome [22]. Several large, prospective, randomized studies have also shown that implantation of implantable cardioverter defibrillator (ICD) improves survivals for those with life-threatening ventricular arrhythmia [3, 23].

Prolonged ambulatory monitoring of patients with unexplained syncope or SCD may identify VPCs. Thus, short-term monitoring may under-detect VPC and leave a substantial proportion of patients unprotected from SCD until such time as VPC is detected. However, prolonged monitoring is expensive and can prove a burden to patients and clinical practices. Thus, identifying those patients who would most benefit from intensive monitoring would be valuable in patients with aborted. Our data indicate that a simple, inexpensive, non-invasive, 10-s test—the AI-enhanced standard ECG—might permit identification of patients with under-detected VPC. Further investigations will be necessary to confirm the diagnostic performance of AI-enabled ECG in specific populations, such as patients with SCD or unexplained syncope and chest tightness, to determine whether AI-enabled ECG could be used to refine the selection of candidates for prolonged ambulatory cardiac rhythm monitoring or to guide treatment in these patients.

### The dimensionality of 12-lead ECG data

While applying CNN analysis in the 12-lead ECG, the one-dimensional approach treats the ECG data as a time-series format. On the other hand, CNN extracts all the features of 12-lead ECG with kernels during two-dimensional data processing. The CNN kernels could be activated by specific wave patterns and recognized by the neural network analysis subsequently [24]. Therefore, two-dimensional analysis is taking the data as an image, more similar to the cardiologist’s way to interpret the 12-lead ECG. However, the two-dimensional data volume is gigantic and much complicated than the one-dimensional data format. Therefore, the general AI tools could not analyze the 12-lead ECG stored with images format [25]. In order to encounter difficulties to analyze these large amounts and complicated two-dimensional data, we used several networks available and different computer architecture combinations to get the best accuracy of VPC prediction by the CNN model. The CNN-based model for VPC prediction from the two-dimensional data was the important feature of this study. This had not been performed successfully before. After optimizing the input model architecture, our two-dimensional CNN model could identify the abnormal ECG and classify the high-risk populations before VPC attacked by the automatic learning paradigm.

From the previous study, the AI-driven algorithms had been applied in automatic diagnosis for various diseases [26], such as myocardial infarction needing urgent revascularization [24], systolic heart failure [25], subtle potassium change among the high-risk populations [26], and atrial fibrillation [25–27]. However, most of these studies were based on the single-lead ECG or one-dimensional (time-series) datasets. From our results, the CNN model derived from the 12-lead ECG and two-dimensional data format was reliable to predict VPC attack automatically and the accuracy was even better than one-dimensional or time-series results (0.895 vs. 0.880). Our study demonstrated the possibility to implement CNN model to identify VPCs using either one-dimensional or two-dimensional data.

### Mechanism by which AI could identify patients with VPC under normal sinus rhythm

The structural changes that underline VPC, which might include myocyte hypertrophy, fibrosis, and chamber enlargement, are likely to lead to subtle ECG changes, allowing for prediction of underlying VPC. This is very similar to using signal average ECG to detect late potentials that could not be observed by human eyes through a single ECG [28, 29]. Furthermore, although seldom reported on ECGs, subtle intraventricular block may correlate with both subtle myocardial fibrosis and risk of VPC or SCD [30]. Thus, it is possible that wavelets on the ECG smaller than the readily observable wave might reflect regional conduction block in these patients. A neural network trained with exposure to plenty of ECGs and with sufficient depth to extract and recall subtle features not routinely appreciated or formally reported by human observers might be powerful enough to identify such features. Finally, it has been reported that AI-enabled ECG may predict left ventricular function [31], and lower left ventricular ejection fraction has been shown as a strong predictor of ventricular arrhythmia [32].

### Limitations

This is one-center study. The results of our observational study may justify future randomized clinical trials for this purpose.

## Conclusions

In this study, the CNN neural network demonstrated as a promising tool for comprehensively human-like interpretation of the ECG. The deep-learning CNN model showed a satisfactory performance in the high-dimensional datasets for the VPC prediction. It will have a great potential deployment in the clinical arena and largely unpredictable implications in the future. However, a key limitation in existing neural networks is explainable. Identifying these features could be of importance because they might offer novel findings that could provide new therapeutic targets or allow for more certainty for clinicians who are otherwise trying to understand what drives the network’s interpretation. Finding ways to peer into this so-called black box is an area of active ongoing investigation.

## Data Availability

All data relevant to this study are included in this manuscript.

## References

[1] Luebbert J, Auberson D, Marchlinski F (2016). Premature ventricular complexes in apparently normal hearts. Card Electrophysiol Clin.

[2] Gorenek B (2020). Premature ventricular complexes: diagnostic and therapeutic considerations in clinical practice: a state-of-the-art review by the American College of Cardiology Electrophysiology Council. J Interv Card Electrophysiol.

[3] Ip JE, Lerman BB (2018). Idiopathic malignant premature ventricular contractions. Trends Cardiovasc Med.

[4] Rincon JA, Guerra-Ojeda S, Carrascosa C, Julian V (2020). An IoT and Fog computing-based monitoring system for cardiovascular patients with automatic ECG classification using deep neural networks. Sensors (Basel).

[5] Simonyan K, Zisserman A. Very deep convolutional networks for large-scale image recognition. arXiv preprint arXiv:1409.1556 2014.

[6] He K, Zhang X, Ren S, Sun J. in European conference on computer vision. 630–645 (Springer).

[7] Szegedy C, Vanhoucke V, Ioffe S, Shlens J, Wojna Z in Proceedings of the IEEE conference on computer vision and pattern recognition. 2818–2826.

[8] Szegedy C, Ioffe S, Vanhoucke V, Alemi AA. in Thirty-first AAAI conference on artificial intelligence.

[9] Chollet F. in Proceedings of the IEEE conference on computer vision and pattern recognition. 1251–1258.

[10] Lin M, Chen Q, Yan S. Network in network. arXiv preprint arXiv:1312.4400 2013.

[11] Hinton GE, Srivastava N, Krizhevsky A, Sutskever I, Salakhutdinov RR. Improving neural networks by preventing co-adaptation of feature detectors. arXiv preprint arXiv:1207.0580 2012.

[12] Ioffe S, Szegedy C. in International conference on machine learning. 448–456 (PMLR).

[13] Xu B, Wang N, Chen T, Li M. Empirical evaluation of rectified activations in convolutional network. arXiv preprint arXiv:1505.00853 2015.

[14] Graham B. Fractional max-pooling. arXiv preprint arXiv:1412.6071 2014.

[15] Bisong E. Building machine learning and deep learning models on Google cloud platform: A comprehensive guide for beginners. 2019.

[16] Abadi, M. *et al.* Tensorflow: Large-scale machine learning on heterogeneous distributed systems. *arXiv preprint *arXiv:1603.04467 2016.

[17] Attia ZI (2019). An artificial intelligence-enabled ECG algorithm for the identification of patients with atrial fibrillation during sinus rhythm: a retrospective analysis of outcome prediction. Lancet.

[18] Wu JT (2017). CHADS(2) and CHA(2)DS(2)-VASc scores predict the risk of ischemic stroke outcome in patients with interatrial block without atrial fibrillation. J Atheroscler Thromb.

[19] Bikkina M, Larson MG, Levy D (1992). Prognostic implications of asymptomatic ventricular arrhythmias: the Framingham Heart Study. Ann Intern Med.

[20] Cohen JD, Neaton JD, Prineas RJ, Daniels KA (1987). Diuretics, serum potassium and ventricular arrhythmias in the Multiple Risk Factor Intervention Trial. Am J Cardiol.

[21] Agarwal SK (2010). Premature ventricular complexes and the risk of incident stroke: the Atherosclerosis Risk In Communities (ARIC) Study. Stroke.

[22] Akkaya M (2013). Efficacy and benefits of catheter ablation of ventricular premature complexes in patients younger and older than 65 years of age. Turk Kardiyol Dern Ars.

[23] Cevik C, Perez-Verdia A, Nugent K (2009). Implantable cardioverter defibrillators and their role in heart failure progression. Europace.

[24] Goto S, Goto S (2019). Application of neural networks to 12-lead electrocardiography- current status and future directions. Circ Rep.

[25] Siontis KC, Noseworthy PA, Attia ZI, Friedman PA (2021). Artificial intelligence-enhanced electrocardiography in cardiovascular disease management. Nat Rev Cardiol.

[26] Feeny AK (2020). Artificial intelligence and machine learning in arrhythmias and cardiac electrophysiology. Circ Arrhythm Electrophysiol.

[27] Ribeiro AH (2020). Automatic diagnosis of the 12-lead ECG using a deep neural network. Nat Commun.

[28] Breithardt G (1993). The signal-averaged ECG: time-domain analysis. Eur Heart J.

[29] Gatzoulis KA (2018). Signal-averaged electrocardiography: past, present, and future. J Arrhythm.

[30] Noureldin RA (2012). The diagnosis of hypertrophic cardiomyopathy by cardiovascular magnetic resonance. J Cardiovasc Magn Reson.

[31] Adedinsewo D (2020). Artificial intelligence-enabled ECG algorithm to identify patients with left ventricular systolic dysfunction presenting to the emergency department with dyspnea. Circ Arrhythm Electrophysiol.

[32] Santangeli P, Rame JE, Birati EY, Marchlinski FE (2017). Management of ventricular arrhythmias in patients with advanced heart failure. J Am Coll Cardiol.

